# LncRNA FLVCR1-AS1 mediates miR-513/YAP1 signaling to promote cell progression, migration, invasion and EMT process in ovarian cancer

**DOI:** 10.1186/s13046-019-1356-z

**Published:** 2019-08-14

**Authors:** Huan Yan, Hong Li, Maria A. Silva, Yichun Guan, Li Yang, Linlin Zhu, Zhan Zhang, Genxia Li, Chenchen Ren

**Affiliations:** 1 Department of Obstetrics and Gynecology, the Third Affiliated Hospital of Zhengzhou University, No. 7 Front Kangfu Street, Zhengzhou, 450052 Henan People’s Republic of China; 2 Department of Pathology and Laboratory Medicine, University of Tennessee Health Science Center, Memphis, TN USA; 3 Center for Reproductive Medicine, the Third Affiliated Hospital of Zhengzhou University, Zhengzhou, Henan People’s Republic of China; 4 Collaborative Innovation Center of Molecular Diagnosis and Laboratory Medicine, Xinxiang Medical University, Xinxiang, Henan People’s Republic of China; 5 Department of Clinical Laboratory, the Third Affiliated Hospital of Zhengzhou University, Zhengzhou, Henan People’s Republic of China

**Keywords:** Ovarian serous cancer, LncRNA, FLVCR1-AS1, EMT, miR-513, YAP1

## Abstract

**Background:**

Long noncoding RNAs (lncRNAs) have been reported to be associated with the proliferation of several cancer cells. The aim of this study was to investigate the role of FLVCR1-AS1 in ovarian serous cancer (OSC).

**Methods:**

FLVCR1-AS1 expression was determined in human OSC tissues, serums and cell lines. The role of FLVCR1-AS1 knockdown or overexpression on OSC cell growth, migration, invasion, apoptosis and epithelial to mesenchymal transition (EMT) were evaluated in vitro using CCK8, colony formation assay, wound healing assay, transwell assay and western blot assay. Besides, luciferase reporter assays were performed to identify interactions among FLVCR1-AS1 and its target genes. Moreover, the in vivo effects were investigated using immunocompromised NSG female mice.

**Results:**

In this study, FLVCR1-AS1 expression was upregulated in OSC tissues, serums, and cells. Knockdown FLVCR1-AS1 decreased cell growth, migration, invasion, and EMT, as well as increased apoptosis in OSC cells, whereas, overexpression of FLVCR1-AS1 increased cell proliferation, migration, invasion, and EMT, and decreased apoptosis of OSC cells. Besides, FLVCR1-AS1 directly bound to miR-513 and downregulated its expression. Moreover, FLVCR1-AS1 reversed the effect of miR-513 on the OSC cell growth, which might be associated with the role of YAP1. Furthermore, in terms of mechanism, FLVCR1-AS1 promoted EMT in OSC cells. Finally, mice models further confirmed that knockdown FLVCR1-AS1 distinctly suppressed cell growth and EMT in vivo.

**Conclusion:**

Taken together, FLVCR1-AS1 mediated miR-513/YAP1 signaling to promote cell progression, migration, invasion and EMT process in OSC cells.

## Background

Ovarian serous cancer (OSC) takes about 85% of ovarian cancer cases, however, the majority of patients are unfortunately diagnosed at an advanced stage, and the median survival rate for OSC is less than 15% [1–4]. In addition, extensive metastases and poor prognosis are common and severe problems in OSC patients, so it is urgent to define its molecular mechanisms of this deadly disease.

Long non-coding RNAs (lncRNAs) play a pivotal role in cancer development, especially in cancer occurrence and metastasis, cell proliferation and apoptosis [5–7]. In OSC, dysfunction of lncRNAs was reported to be associated with cell progression and metastasis. For example, previous study showed that lncRNA DQ786243 was upregulated in OSC cells, and knockdown of DQ786243 inhibited cell progression of via regulating miR-506 [8]. Besides, lncRNA MLK7-AS1 promoted migration of OSC cells via miR-375/YAP1 axis [9]. Moreover, Lin28A regulated the survival, invasion, metastasis, and apoptosis through ROCK2 in OSC cells [10]. However, the molecular function of lncRNAs in OSC remains largely unknown.

FLVCR1-AS1 is recently discovered upregulated in hepatocellular cancer, gastric cancer and lung cancer [11–14]. However, few studies have studied FLVCR1-AS1 in OSC. In this study, FLVCR1-AS1 expression was elevated in OSC tissues and cell lines. Consistent with previous studies, we found that FLVCR1-AS1 promoted cell proliferation, colony formation, migration and invasion, while inhibited cell apoptosis in OSC. Besides, FLVCR1-AS1 increased cell progression of OSC by interacting with miR-513 to upregulate expression of YAP1. Moreover, mouse xenograft model further confirmed that knockdown FLVCR1-AS1 suppressed tumor growth in vivo.

The occurrence of epithelial-mesenchymal transition (EMT) causes losing biological characteristics in epithelial cells, but obtaining features of mesenchymal cells. Several studies reported lncRNA was involved in EMT process [15–17]. In our study, knockdown of FLVCR1-AS1 inhibited EMT process, while FLVCR1-AS1 overexpression promoted EMT process in ovarian cancer cells, which was also confirmed in vivo. In sum, these findings revealed for the first time that FLVCR1-AS1 /miR-513/YAP1 axis plays a role in OSC cells.

## Materials and methods

### Patients samples

50 OSC tumor tissues, adjacent normal tissues, and serum samples were collected from patients between Mar 2016 and Oct 2018 at the third affiliated Hospital of Zhengzhou University. All patients wrote the informed consents, and the study was approved by the local ethics committee (no.2016–56)

### Cell culture and transfection

All cell lines were purchased from ATCC. The small-interfering RNA (siRNA) for FLVCR1-AS1 and YAP1, overexpression plasmids for FLVCR1-AS1-pcDNA 3.1, miR-513 promoter/inhibitor were designed by GenePharma. The sequences for FLVCR1-AS1 were as follows: FLVCR1-AS1–1: 5′-CAGGAAAATGTCAGCCAGCG-3′; FLVCR1-AS1–2: 5′-GCCTCTAAGTAGTGACACTA-3′; and the siRNA sequence that targeted FLVCR1-AS1 for knockdown was si-FLVCR1-AS1–1:5′-GGTAAGCAGTGGCTCCTCTAA-3′, si-FLVCR1-AS1–2:5′-CGCTTAACAGCTAAGCGCATA-3′.

The sequence for YAP1 was 5′-ATCTCTGACTGATTCTCTGGC-3′; and the siRNA sequence that targeted YAP1 was:5′-CGGCAGGTCCTCAACCTGAAT-3′ .

### Quantitative real-time PCR (qRT-PCR)

To investigate FLVCR1-AS1 expression in tissue samples and serums, qRT-PCR was applied on the Roche Lightcycler 480 RT-PCR system. The extraction of total RNA from tissue and cell samples was performed using TRIzol reagent (Invitrogen, Carlsbad, CA), while RNA in serum was done with Qiagen miRNeasy Serum Kit (Hilden, Germany). Then RNA samples were used for synthesis of cDNA. All of the specimens were tested in triplicate.

### Cell counting kit-8(CCK-8)

The cell proliferation was determined using CCK-8. After incubation for 24, 48, 72, and 96 h, 15ul of CCK-8 reagent was added and determined at a wavelength of 450 nm.

### Cell Colony formation assay

OSC cells (800 cells/plate) were put into 6 well plates after 48 h transfection, and incubated in medium for 21 days, and then the plates were stained with 0.5% crystal violet (Santa Cruz, Dallas, TX, USA).

### Soft agar Colony formation assay

Firstly, 6 well plates were prepared by 0.6% agarose in growth medium, 20 min later, OSC cells (200 per well) in 0.4% agarose were placed on the medium, then add 1 ml growth medium into each well each 3 days. After 21 days, the colonies were counted with three random fields under a digital microscopy.

### Transwell migration and invasion assay

The assays were performed using 24-well transwell chambers with (migration) or without (invasion) Matrigel. The concentration of medium on the upper and lower chamber was serum-free and with 10% FBS, respectively. The wells were fixed and stained after a day. Then stained cells were counted with five random fields.

### Luciferase reporter assay

The 3′-UTR sequence of miR-513 binding sites within the predicted target sites was amplified and cloned into the pmirGLO luciferase vector. SKOV3 cells co-transfected with the pmirGLO-WT or -MUT or miR-513 mimics/NC or inhibiror/NC using Lipo2000. After 48 h, the luciferase activity was analyzed with the Dual-luciferase reporter assay system.

### Western blot assay

The tissues and cells proteins were extracted and the concentrations were measured. Protein samples were separated and transferred onto a membrane and then incubated with primary antibodies (1:1000, Abcam) at 4 °C overnight and then with secondary antibodies for 1 h. All the bands were determined using ECL detection system.

### Immonofluorescence staining assay

OSC cells were fixed with 4% paraformaldehyde and incubated with 5% goat serum, 3% BSA, and 0.1% Triton-X100. Primary antibodies E-cadherin and vimentin (1:200; Abcam) were incubated with SKOV3 cells for 12 h. Cell nuclei were stained with DAPI (CA, CST).

### Xenograft mouse model

The animal study was approved by the Animal research Committee of our hospital. Female NSG mice (6-week) were injected with SKOV3 cells transfected with FLVCR1-AS1 siRNA/controls, respectively. 4 weeks after injection, the tumors were collected and measured.

### Statistical analysis

The results are shown as mean ± SD. Statistical analysis was performed using SPSS 20.0. Differences were compared using t-test or oneway ANOVA. In the bar graphs, * indicates *P* < 0.05.

## Results

### FLVCR1-AS1 expression is upregulated in OSC tissues, serums, and cell lines and predicted poor prognosis

**Fig. 1:**
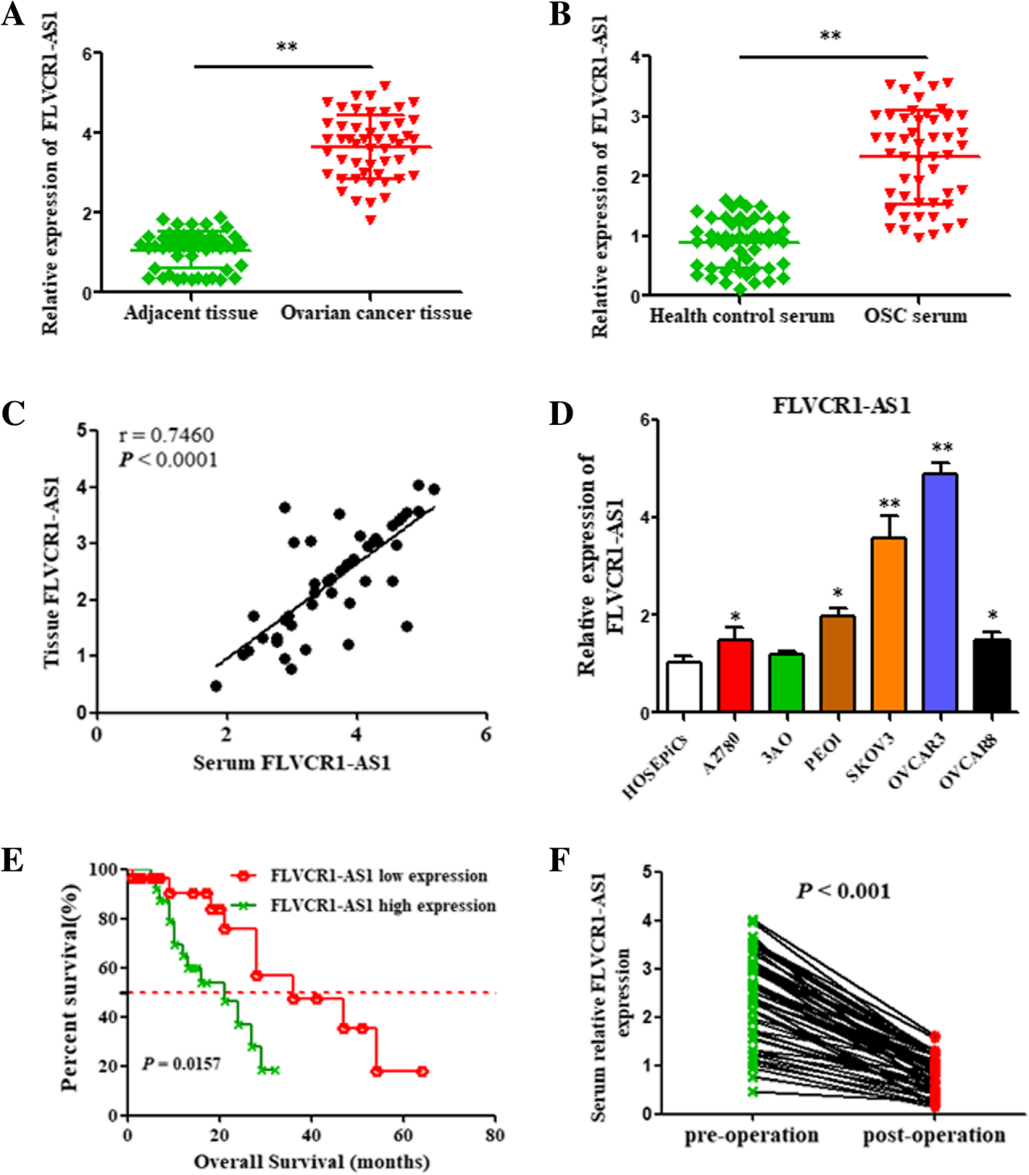
FLVCR1-AS1 is upregulated in OSC tissues, serum, and cell lines and predicted poor prognosis in OSC. **a** Expression levels of FLVCR1-AS1 were upregulated in ovarian cancer tissues compared with adjacent nontumor tissues. **b** Expression levels of FLVCR1-AS1 were upregulated in ovarian cancer serum samples compared with healthy controls. **c** Correlation of FLVCR1-AS1 expression levels in ovarian cancer tissue and serum. **d** Expression levels of FLVCR1-AS1 in OSC cell lines compared to the normal human ovarian surface epithelial cells. **e** The overall survival (OS) rate was significantly lower in patients with high FLVCR1-AS1 expression than in those with low FLVCR1-AS1 expression. **f** Serum FLVCR1-AS1 expression levels were downregulated in post-operation samples compared with pre-operation ones. Data presented as the mean ± standard deviation (SD) of three independent experiments. *P < 0.05, **P < 0.01

Results showed that FLVCR1-AS1 expression was upregulated in OSC tissues compared with adjacent non-tumor tissues (Fig. 1a, P < 0.01). Besides, FLVCR1-AS1 expression was also increased in the serum of OSC patients compared with health controls (Fig. 1b, P < 0.01), and a positive association was observed between mRNA levels of FLVCR1-AS1 in OSC serum and tissues (Fig. 1c; r^2^ = 0.7460, *P* < 0.0001). FLVCR1-AS1 expression levels were elevated in OSC cell lines (especially SKOV3 and OVCAR3 cell lines) than normal ovarian epithelial cells (Fig. 1d; *P* < 0.05). Moreover, according to FLVCR1-AS1 mRNA expression, we divided 50 OSC patients into a relatively high FLVCR1-AS1 level group (*n* = 27, 1.5-fold higher than normal tissues) and relatively low FLVCR1-AS1 level group (*n* = 23). Noticeably, higher FLVCR1-AS1 expression in OSC was significantly correlated with lymphatic metastasis and distant metastasis (*P* < 0.001) (Table 1). To determine the association with clinical survival of OSC patients, overall survival (OS) analysis were performed in this study. Patients with high FLVCR1-AS1 level had poorer OS rates than those with low FLVCR1-AS1 level ones (Fig. 1e; *P* = 0.0157). In addition, FLVCR1-AS1 expressions in postoperative serum samples were downregulated than those in preoperative ones (*n* = 50) (Fig. 1f; P < 0.001), which suggested that high FLVCR1-AS1 expression was related with poor prognosis of OSC patients.

**Table 1:** Relationship between clinicopathological factors and FLVCR1-AS1 expression in OSC patients

		Total number	FLVCR1-AS1 expression				
Characteristics			High		Low		*P*-value
			n	%	n	%	
Age	< 55	20	11	55	9	45	0.349
	≥55	30	16	53.3	14	46.7	
CA125	< 35	24	13	54.2	11	45.8	0.403
	≥35	26	14	53.8	12	46.2	
Lymph node metastasis	+	36	23	63.9	13	36.1	< 0.001
	–	14	4	28.6	10	71.4	
Distant metastasis	+	37	25	67.6	12	32.4	< 0.001
	–	13	2	15.4	11	84.6	

### FLVCR1-AS1 promoted cell proliferation and inhibited apoptosis abilities in OSC cells

**Fig. 2:**
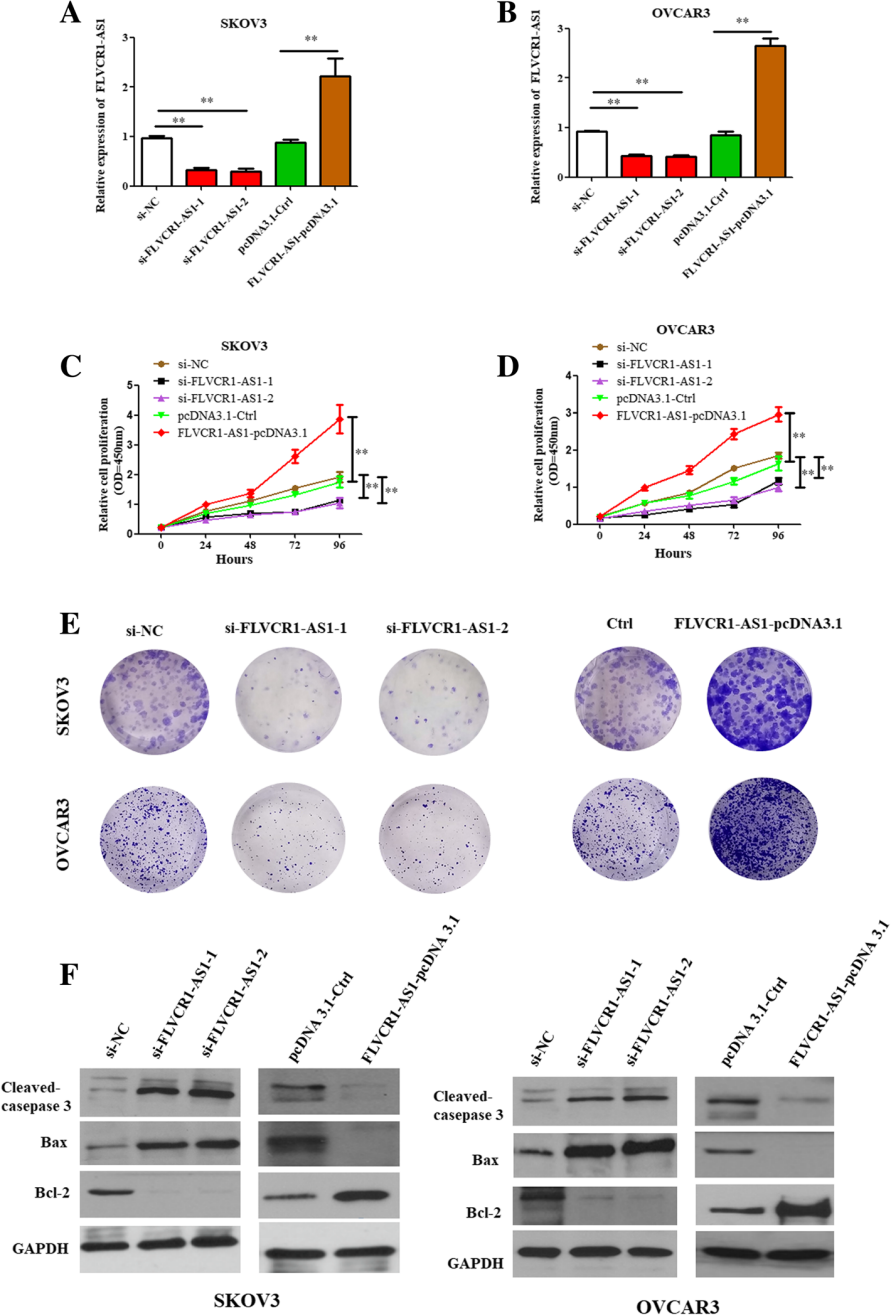
FLVCR1-AS1 promoted cell proliferation and inhibited cell apoptosis abilities in OSC cells. **a**, **b** The transfection efficiency of OSC cells in response to transfection with FLVCR1-AS1 si-RNA or overexpression plasmid. FLVCR1-AS1 expression was inhibited when FLVCR1-AS1 was knockdown, while was promoted when FLVCR1-AS1 was overexpressed in both cell lines. **c**, **d** Cell proliferation was assayed in SKOV3 and OVCAR3 cells transfected with FLVCR1-AS1 si-RNA or overexpression plasmid using CCK8 at 0 h, 24 h, 48 h, 72 h and 96 h time point. **e** Colony formation assays were performed after transfection with FLVCR1-AS1 si-RNA or overexpression plasmid in both cell lines. **f** Apoptosis marker cleaved-caspase 3, Bax, and Bcl-2 were detected using western blot assay in SKOV3 and OVCAR3 cells transfected with FLVCR1-AS1 si-RNA and overexpression plasmid. *P < 0.05, **P < 0.01

As shown in Fig. 2a and b, FLVCR1-AS1 expression was decreased in si-FLVCR1-AS1 transfected cells than in the control cells, while increased in FLVCR1-AS1-overexpression plasmid transfected cells compared with the controls in both OSC cell lines.

CCK8 assay showed that cell proliferation was inhibited when FLVCR1-AS1 was knockdown, while was promoted when FLVCR1-AS1 was overexpressed in both OSC cells (Fig. 2c and d; *P* < 0.01). Similar results were found in colony formation abilities of OSC cells (Fig. 2e). Moreover, apoptosis results revealed knockdown FLVCR1-AS1 induced the apoptosis ability in OSC cells, while the apoptosis ability was suppressed when FLVCR1-AS1 was overexpressed (Fig. 2f). Taken together, FLVCR1-AS1 promoted proliferation and inhibited apoptosis abilities in OSC cells.

### FLVCR1-AS1 promoted cell migration and invasion abilities in OSC cells

**Fig. 3:**
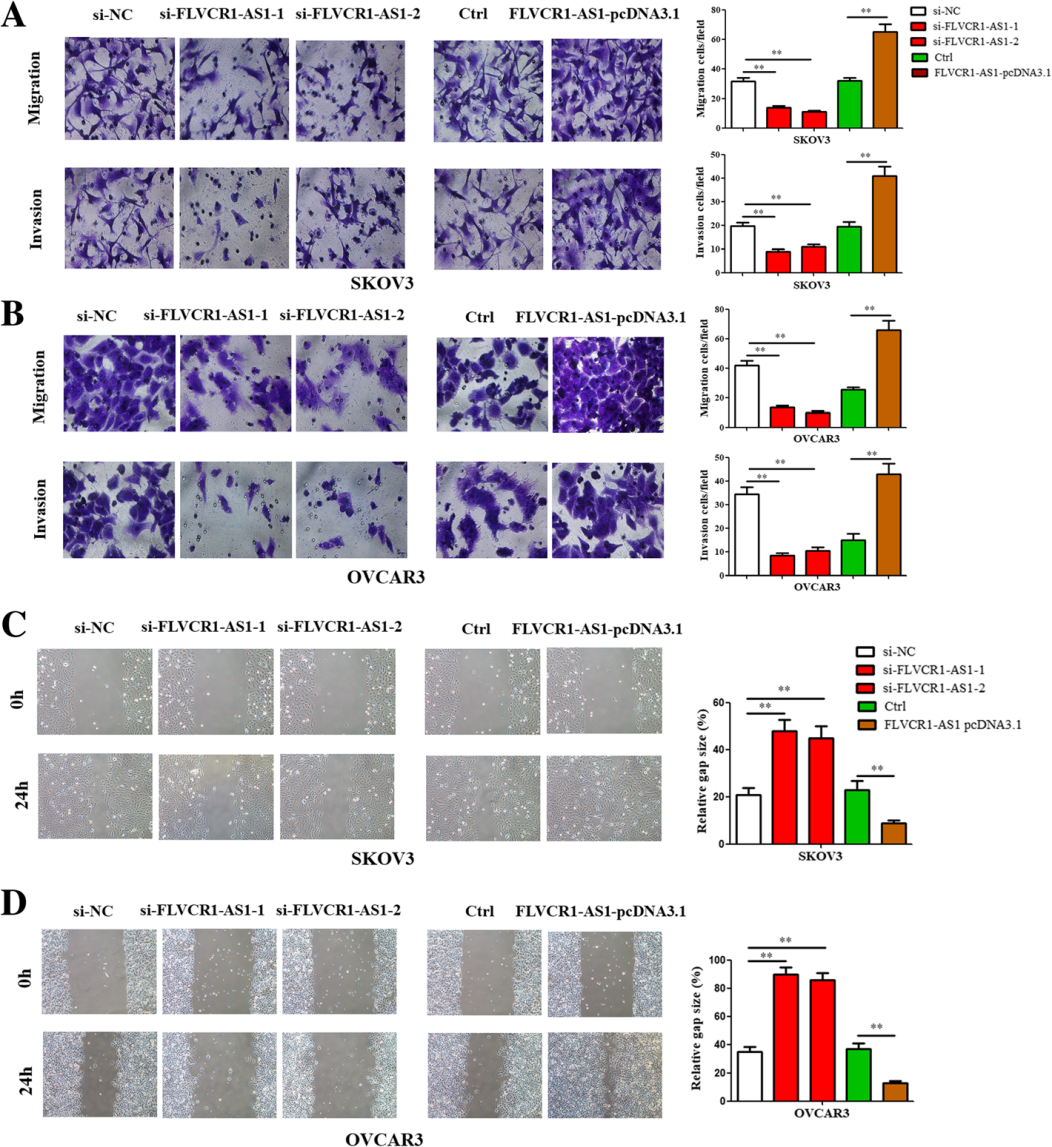
FLVCR1-AS1 promoted cell migration, invasion and wound healing in OSC cells. **a**, **b** Transwell assays were used to measure the effect of FLVCR1-AS1 knockdown or overexpression on cell migration and invasion in SKOV3 and OVCAR3 cells (magnification 400 ×). **c**, **d** Wound healing assay was performed in both cell lines following transfection with FLVCR1-AS1 si-RNA or overexpression plasmid (magnification 100 ×). The data were presented as mean ± SD of three independent experiments. The statistical results were shown on the right panel. **P < 0.01

Results showed that knockdown FLVCR1-AS1 suppressed OSC cells migration and invasion ability, while FLVCR1-AS1 overexpression promoted the ability in both two cells (Fig. 3a and b; *P* < 0.01). Similarly, the wound healing ability in both cell lines was impaired when FLVCR1-AS1 knockdown compared to the si-NC group, while the wound healing ability was obviously repaired in the FLVCR1-AS1 overexpression group than control group (Fig. 3c and d; *P* < 0.01).

### FLVCR1-AS1 regulated miR-513 expression in OSC cells

**Fig. 4:**
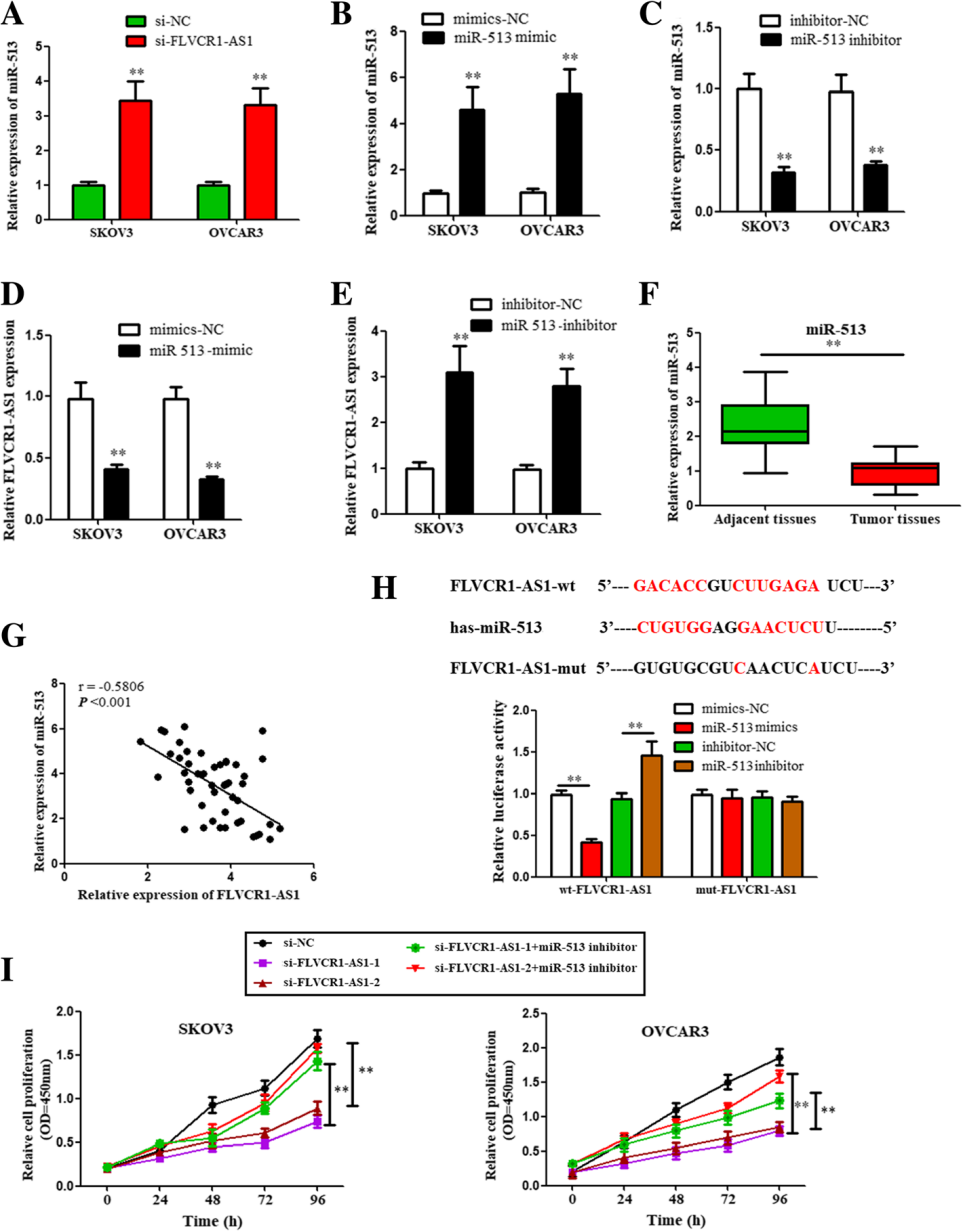
FLVCR1-AS1 regulated miR-513 expression in OSC cells. **a** miR-513 expression was significantly upregulated after FLVCR1-AS1 knockdown. **b**, **c** miR-513 overexpression and inhibition were transfected into SKOV3 and OVCAR3 cells, and the transfection efficiency was verified using qRT-PCR. **d**, **e** FLVCR1-AS1 mRNA expression was significantly decreased in response to miR-513 overexpression while increased in response to miR-513 inhibition. **f** miR-513 expression levels were significantly decreased in human OSC tissues compared with adjacent normal tissues. **g** An inverse correlation was observed between mRNA expressions of miR-513 and FLVCR1-AS1 in human OSC tissues. **h** FLVCR1-AS1 interacted with miR-513 by directly targeting. **i** CCK8 results showed that FLVCR1-AS1 were significantly downregulated in FLVCR1-AS1 knockdown groups compared to the si-NC group, while the effects were reversed by miR-513 inhibition cotransfected groups. The data were presented as mean ± SD of three independent experiments. **P < 0.01

Bioinformatics analysis was performed to predict FLVCR1-AS1-targeted miRNAs using miRDB (http://mirdb.org/miRDB/) and lncBase (http://carolina.imis.athena-innovation.gr/diana_tools/web/index.php?r=lncbasev2%2Findex-predicted). To determine whether FLVCR1-AS1 regulated miR-513 expression in cell progression of OSC cells, correlation between FLVCR1-AS1 and miR-513 were detected. qRT-PCR results showed that miR-513 expression was upregulated when FLVCR1-AS1 was knockdown (Fig. 4a; *P* < 0.01). Then, transfection effects of miR-513 overexpression or inhibition were verified using qRT-PCR in both cells (Fig. 4b and c; *P* < 0.01). FLVCR1-AS1 expression was decreased after miR-513 was overexpressed while increased after miR-513 was inhibited (Fig. 4d and e; *P* < 0.01). Besides, miR-513 expressions were suppressed in OSC tissues than adjacent non-tumor tissues (Fig. 4f; *P* < 0.01). In addition, an inverse correlation was found between the mRNA expressions (Fig. 4g; *P* < 0.001). A dual-luciferase reporter assay confirmed the binding site between FLVCR1-AS1 and miR-513 in our study (Fig. 4h; *P* < 0.01). Furthermore, CCK8 results showed that the downregulation of OSC cell proliferation caused by inhibiting FLVCR1-AS1 was reversed by miR-513 inhibitor (Fig. 4i; P < 0.01). Therefore, FLVCR1-AS1 might promote cell growth through down regulating the activity of miR-513 in OSC cells.

### YAP1 promoted cell progression in OSC cells

**Fig. 5:**
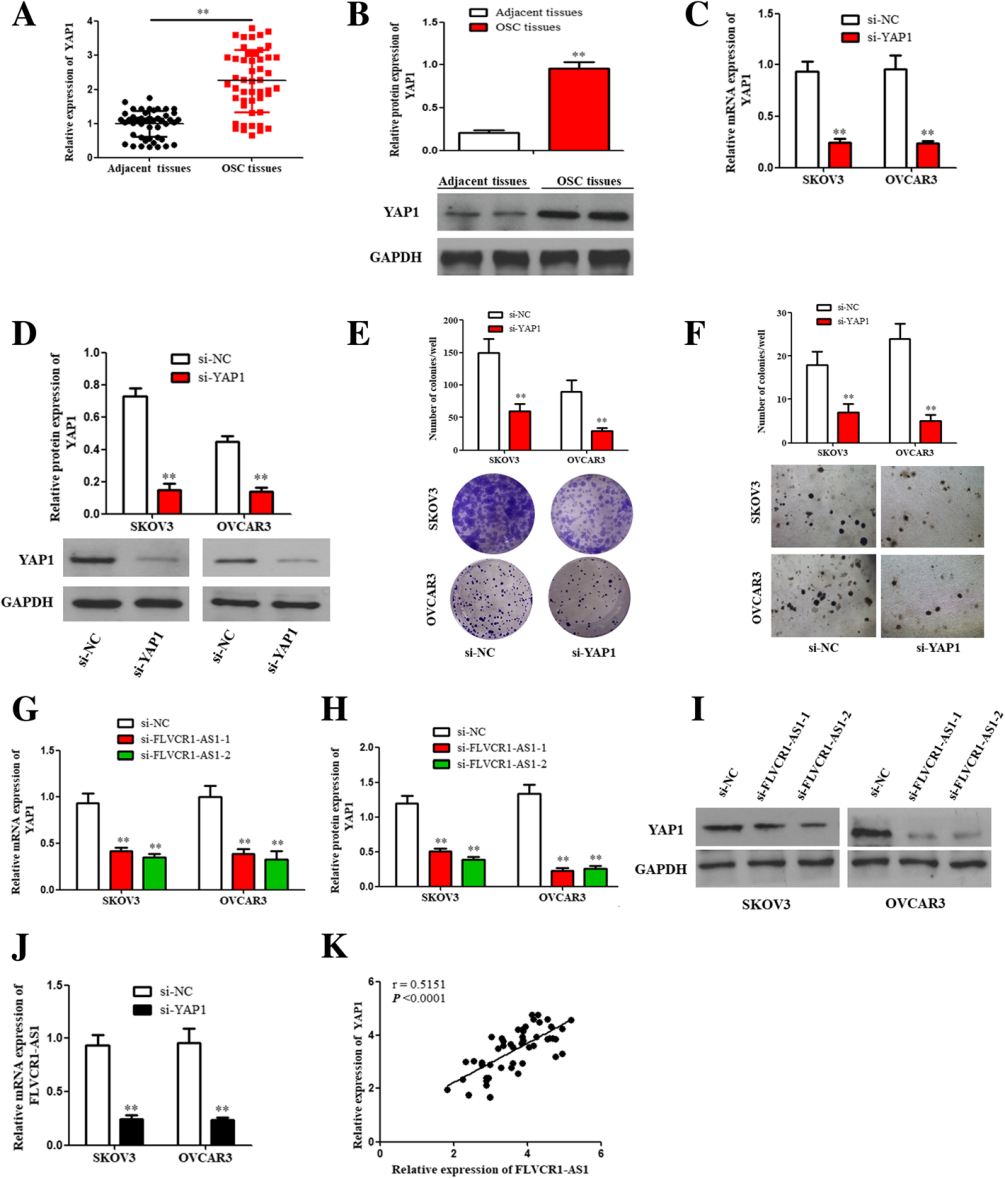
The expression levels of YAP1 and the correlation between FLVCR1-AS1 and YAP1 in OSC cells. **a**, **b** The mRNA and protein expression levels of YAP1 were significantly elevated in OSC tissues compared with adjacent normal tissues. **c**, **d** YAP1 si-RNA was transfected into SKOV3 and OVCAR3 cells, and the transfection efficiency was verified using qRT-PCR and western blot assays. **e**, **f** Colony formation and soft agar growth assays revealed that the colonies generated of both cells were decreased when YAP1 expression was inhibition. **g**, **h**, **i** YAP1 protein and mRNA expression levels were greatly reduced by FLVCR1-AS1 knockdown in OSC cell lines. **j** FLVCR1-AS1 mRNA expression was reduced following YAP1 inhibition in both cell lines. **k** A positive correlation was observed between FLVCR1-AS1 and YAP1 mRNA expressions in human OSC tissues. The data were presented as mean ± SD of three independent experiments. **P < 0.01

Results showed that YAP1 were elevated in OSC tissue than adjacent normal tissues (Fig. 5a and b; *P* < 0.01). YAP1 knockdown was transfected in OSC cells, and was verified using qRT-PCR and western blot assay (Fig. 5c and d; P < 0.01). Colony formation and soft agar growth assays showed that colonies were decreased when YAP1 was inhibited in both cells, which revealed that YAP1 promoted progression of OSC cells (Fig. 5e and f; *P* < 0.01).

### The correlation between FLVCR1-AS1 and YAP1 in OSC cells

Results showed that YAP1 expression was decreased by FLVCR1-AS knockdown in both cells (Fig. 5g, h and i; P < 0.01); Besides, FLVCR1-AS1 expression was suppressed following YAP1 knockdown in OSC cells (Fig. 5j; *P* < 0.01). Furthermore, a positive correlation was found between FLVCR1-AS1 and YAP1 mRNA expressions in OSC tissues (Fig. 5k; *P* < 0.001).

### FLVCR1-AS1 directly regulated YAP1 in OSC cell growth

**Fig. 6:**
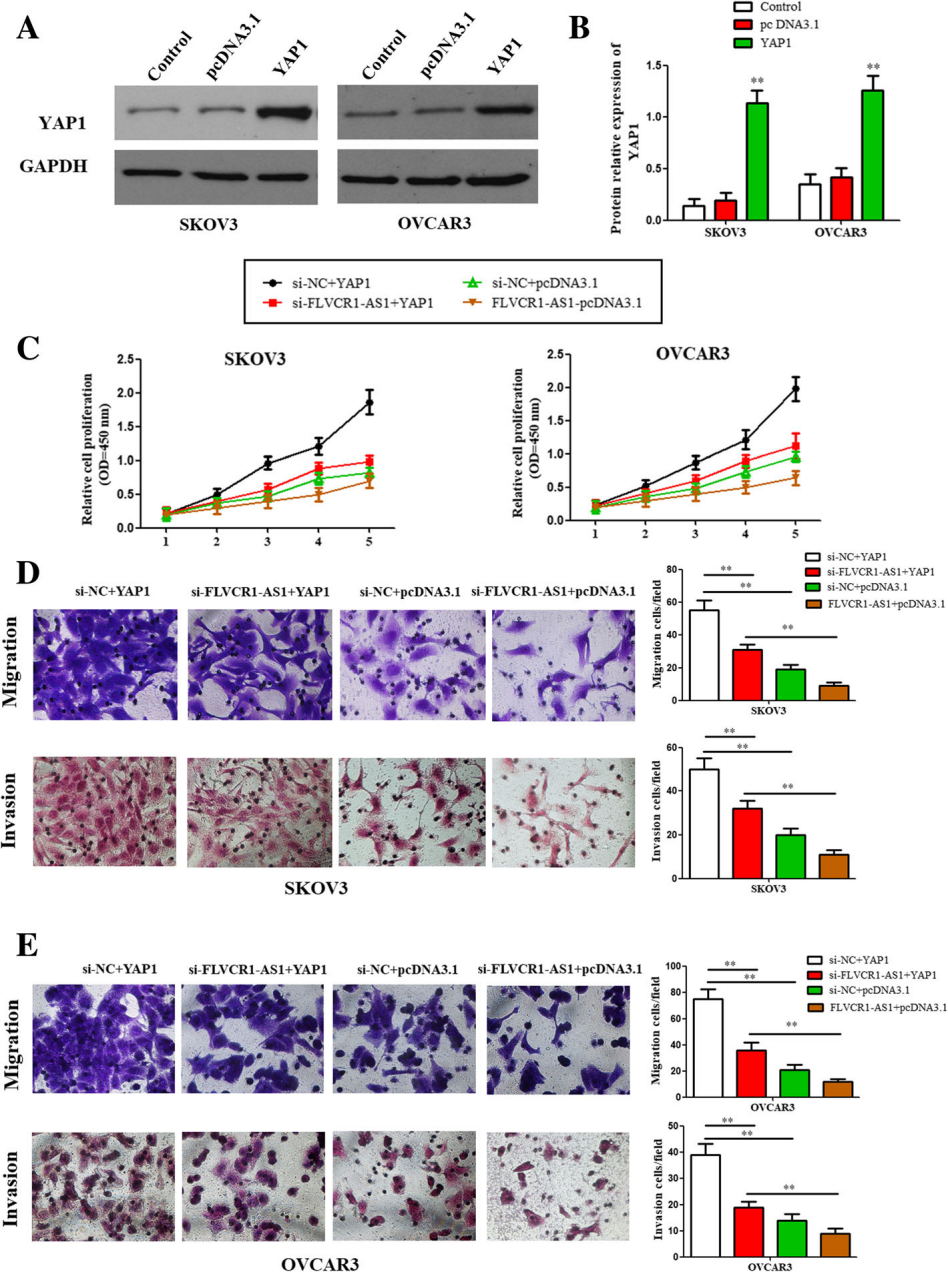
FLVCR1-AS1 regulated cell proliferation, migration and invasion through targeting YAP1 in OSC cells. **a**, **b** The plasmids of pcDNA-3.1/YAP1 were transfected into SKOV3 and OVCAR3 cell lines to achieve YAP1 overexpression. The transfection efficiency was verified using western blot assay. **c** Si-NC/si-FLVCR1-AS1 and pcDNA3.1/YAP1 were cotransfected into SKOV3 and OVCAR3 cell lines. CCK8 assay was used to determine the cell growth. **d**, **e** The cell migration and invasion in both SKOV3 and OVCAR3 cell lines were significantly reduced by FLVCR1-AS1 knockdown, while were partly restored in response to YAP1 overexpression (magnification 400×). The data were presented as mean ± SD of three independent experiments. **P < 0.01

The transfection efficiency of pcDNA-3.1/YAP1 plasmids in both cells was verified using western blot assay (Fig. 6a and b). CCK8 results showed that cell proliferation was decreased when FLVCR1-AS1 were knockdown, while increased when YAP1 were overexpression; and the effect after FLVCR1-AS1 knockdown on OSC cell growth was reversed by YAP1 overexpression (Fig. 6c; *P* < 0.01). Similar results were found in migration and invasion assays (Fig. 6d and e, P < 0.01). In short, FLVCR1-AS1 might regulate cell growth, migration and invasion via targeting YAP1 in OSC cells.

### miR-513 negatively regulated YAP1 expression in OSC cell

**Fig. 7:**
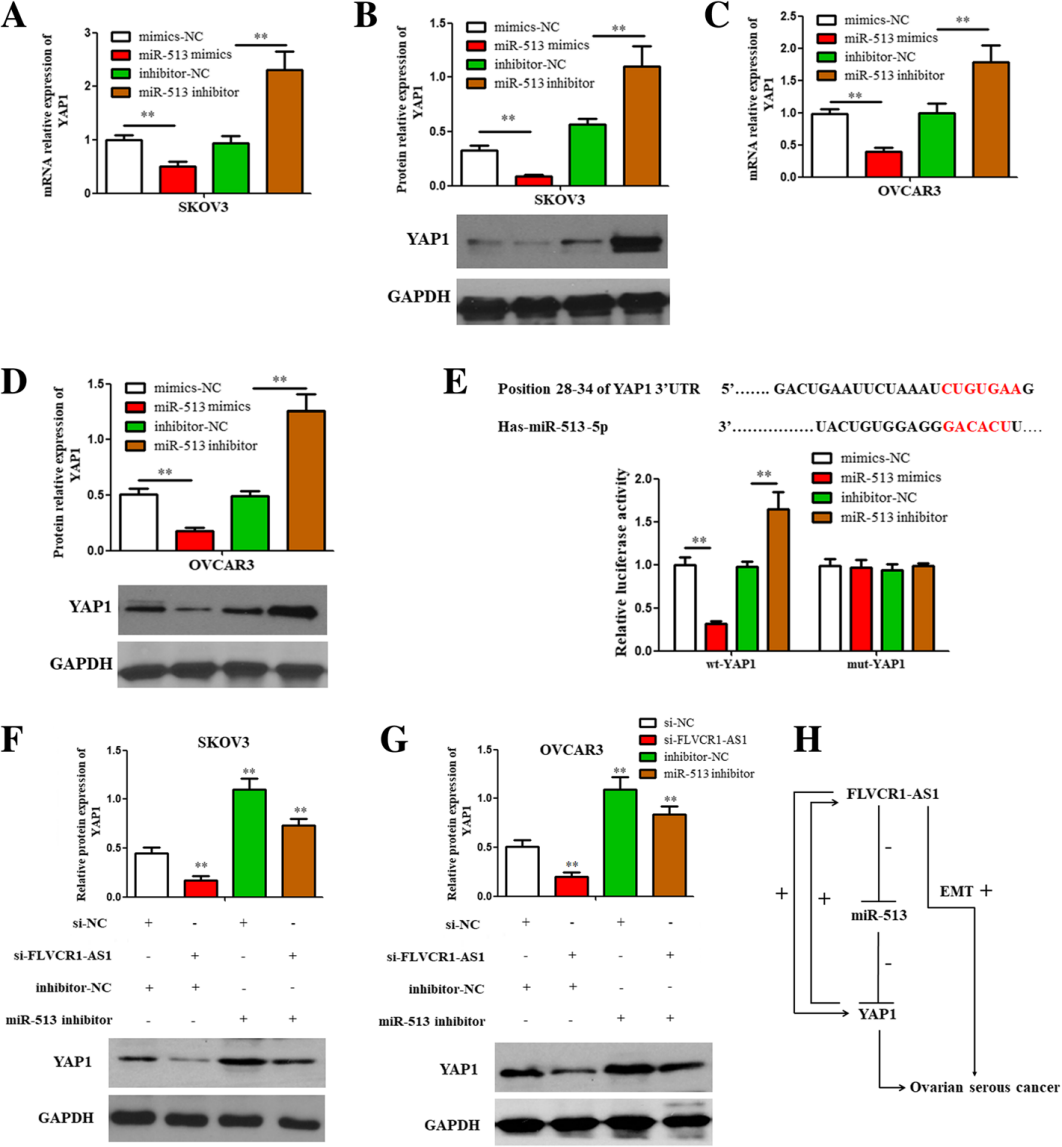
FLVCR1-AS1 regulated YAP1 expression via miR-513 in OSC cells. **a**, **b**, **c**, **d** qRT-PCR and western blot assay showed that mRNA and protein expression levels of YAP1 were downregulated following miR-513 overexpression while upregulated following miR-513 inhibition in both cell lines. **e** miR-513 negatively regulated YAP1 by targeting to its 3’UTR. **f**, **g** The inhibitory function of FLVCR1-AS1 knockdown on YAP1 protein level was partially restored by miR-513 inhibitor. **h** FLVCR1-AS1 regulated YAP1 level through miR-513 in OSC cells. The data were presented as mean ± SD of three independent experiments. **P < 0.01

Results showed that YAP1 expression was decreased when miR-513 was overexpressed while increased when miR-513 was inhibited in both two cells (Fig. 7a, b, c and d; *P* < 0.01). Similarly, a dual-luciferase reporter assay further confirmed the binding site (Fig. 7e; *P* < 0.01). In sum, the results indicated that miR-513 directly regulated YAP1 in OSC cell.

### FLVCR1-AS1 regulated YAP1 level through miR-513

Western blot result showed that YAP1 protein expressions were decreased in response to si-FLVCR1-AS1 transfection, while were significantly increased following miR-513 inhibition in both two cells (Fig. 7f and g; P < 0.01), which suggested that the inhibition effect induced by FLVCR1-AS1 knockdown on YAP1 protein expression was reversed by miR-513 inhibition (Fig. 7h).

### FLVCR1-AS1 promoted EMT in OSC cell

**Fig. 8:**
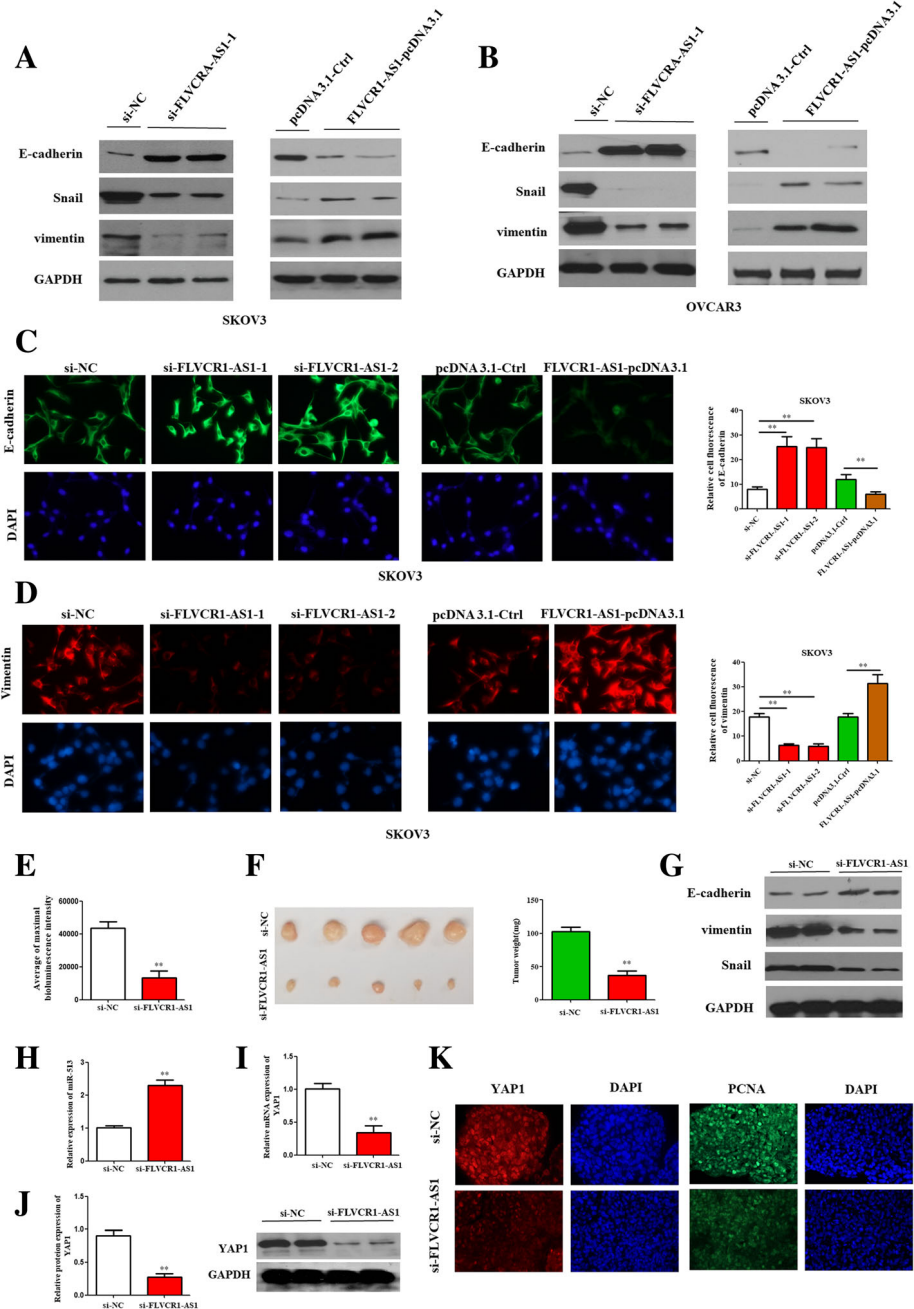
FLVCR1-AS1 promoted EMT process and in vivo tumor growth in OSC cells. **a**, **b** FLVCR1-AS1 knockdown significantly promoted the expression of E-cadherin but inhibited Snail and vimentin, whereas FLVCR1-AS1-overexpression led to the opposite results in OSC cells compared with control group. **c**, **d** The positive immunostaining SKOV3 cells of E-cadherin were increased, while vimentin was decreased when FLVCR1-AS1 was knockdown compared to the si-NC group; however, FLVCR1-AS1-overexpression led to the opposite results compared with controls in OSC cells. **e** Bioluminescence of Xenograft live imaging of mice at 4 weeks following intrabursal injection of si-FLVCR1-AS1 or si-NC transfected SKOV3 cells. **f** Tumor weight in mice primary ovaries injected with si-FLVCR1-AS1 or si-NC SKOV3 cells. Inhibition of FLVCR1-AS1 inhibits OSC cell tumor growth in vivo. **g** FLVCR1-AS1 knockdown significantly promoted the expression of E-cadherin but inhibited Snail and vimentin in mice tumors. **h** The mRNA expression of miR-513 in mice primary tumors injected with si-FLVCR1-AS1 or si-NC SKOV3 cells. **i**, **j** mRNA and protein expressions of YAP1 in mice primary tumors injected with si-FLVCR1-AS1 or si-NC SKOV3 cells. **k** The positive immunostaining cells of YAP1 and PCNA were decreased following FLVCR1-AS1 knockdown compared to the negative control group in mice primary tumors of ovary. **P < 0.01

In terms of mechanisms, protein expressions of vimentin and snail were downregulated, whereas E-cadherin expression was upregulated in si-FLVCR1-AS1 transfected group compared with si-NC group. Whereas, opposite results were found in FLVCR1-AS1 overexpression transfected OSC cells compared to the controls (Fig. 8a and b; *P* < 0.01). Moreover, immunofluorescent staining results showed that E-cadherin was strongly stained in si-FLVCR1-AS1 transfected SKOV3 cells compared to si-NC transfected cells, whereas vimentin was weekly stained following FLVCR1-AS1 knockdown compared to si-NC transfected cells. Similarly, opposite results were found in FLVCR1-AS1 overexpression transfected SKOV3 cells compared to the controls (Fig. 8c and d). FLVCR1-AS1 might regulate EMT markers and promote EMT process in OSC cells (Fig. 7h).

### FLVCR1-AS1 knockdown suppressed tumor growth and EMT in vivo

FLVCR1-AS1 knockdown obviously suppressed tumor growth and weight in xenograft mouse models (Fig. 8e, and f; P < 0.01). EMT marker genes in mice tumors were examined (Fig. 8g; P < 0.01), results showed that FLVCR1-AS1 knockdown significantly promoted E-cadherin expression but inhibited vimentin and Snail expressions in the FLVCR1-AS1 knockdown mice tumors than the control mice, which were consistent with the results in vitro. Besides, to determine whether FLVCR1-AS1 regulated OSC cells proliferation through miR-513/YAP1 in vivo, miR-513 and YAP1 expressions were detected in mice primary tumors of ovary. miR-513 mRNA expression was increased in mice primary tumors injected with si-FLVCR1-AS1 compared with si-NC SKOV3 cells injected group (Fig. 8h; P < 0.01). Furthermore, mRNA and protein expressions of YAP1 were decreased in mice primary tumors injected with si-FLVCR1-AS1 compared with si-NC SKOV3 cells injected group (Fig. 8i and j; P < 0.01). Moreover, the positive immunostaining cells of YAP1 and PCNA were decreased following FLVCR1-AS1 knockdown compared to the control group in mice primary tumors of ovary (Fig. 8k; P < 0.01). In sum, FLVCR1-AS1 knockdown suppressed tumor growth through miR-513/YAP1 signaling in vivo and FLVCR1-AS1 might be an oncogene in OSC cells.

## Discussion

FLVCR1-AS1 expression has been reported to be upregulated in lung gastric and hepatocellular cancers [11–14]. Similarly, elevated FLVCR1-AS1 expressions were found in OSC tissues, serums, and cell lines in the current study. Besides, higher FLVCR1-AS1 expression was related with poor prognosis in OSC patients. Consistent with Zhang et al., Yan et al., and Gao et al., knockdown FLVCR1-AS1 expression inhibited OSC cell growth, migration, and invasion, as well as promoted cell apoptosis, while overexpressing of FLVCR1-AS1 led to the opposite results. In addition, knockdown of FLVCR1-AS1 suppressed OSC tumor growth in vivo, which illustrated that FLVCR1-AS1 might be a promoter in OSC cell progression.

Studies have indicated that miR-513 played antitumor effects in several human tumors [18, 19]. Similarly, miR-513 expression was decreased in OSC tissues. Besides, miR-513 inversely regulated FLVCR1-AS1 expression in OSC tissues. In the meantime, a luciferase assay further confirmed the binding between them. Taken together, FLVCR1-AS1 might suppress miR-513 through regulating its downstream target mRNAs in OSC.

As we know, Hippo pathway, especially the dysfunction of YAP1 plays tumor-promoting actions in several human cancers [20–24]. Our previous findings demonstrated that YAP1 expression was upregulated in OSC cells [8], which were confirmed in this study. YAP1 protein expression was decreased in response to FLVCR1-AS1 knockdown. Besides, when si-FLVCR1-AS1 and YAP1 overexpression plasmid were co-transfected into OSC cells, the inhibitory effect of si-FLVCR1-AS1 on OSC cell proliferation, migration and invasion were by YAP1 overexpression. FLVCR1-AS1 might regulate YAP1 expression to modulate proliferation, migration and invasion in OSC cells. Moreover, YAP1 mRNA and protein expression were inversely regulated by miR-513 in both OSC cells, and a luciferase assay further confirmed the direct binding of miR-513 to YAP1, which proved that YAP1 was a target gene of miR-513.

Since we confirmed that FLVCR1-AS1 interacted with YAP1 to regulate OSC cell growth, and we have proved the direct binding between FLVCR1-AS1 and miR-513 as well as between miR-513 and YAP1 in OSC cells, we presumed that FLVCR1-AS1 might regulate YAP1 expression via miR-513. Western blot assay showed that YAP1 protein level was decreased following FLVCR1-AS1 knockdown, while was increased following miR-513 inhibition. Furthermore, the effect of silencing FLVCR1-AS1 on YAP1 was restored following miR-513 inhibition. Therefore, FLVCR1-AS1 regulated YAP1 through miR-513 in OSC cells.

To detect the role of FLVCR1-AS1 in regulating EMT process, E-cadherin, vimentin, and Snail expressions in OSC cells were determined using western blot and immunofluorescence assays. Interestingly, decreased E-cadherin, increased expressions of vimentin and Snail expressions were found in the FLVCR1-AS1 knockdown group than si-NC group, while decreased expression levels of vimentin and Snail, increased expressions of E-cadherin were found in the FLVCR1-AS1 overexpression group compared to the control group both in vitro and *vivo*, similar results were found in immunofluorescence assay, suggesting that FLVCR1-AS1 played a promotion role in EMT in OSC cells.

## Conclusions

In summary, FLVCR1-AS1/miR-513/YAP1 axis plays a vital role in the cell growth, migration, invasion, tumorigenesis, and EMT in OSC cells, which indicated that FLVCR1-AS1 could act as a potential therapeutic target for human ovarian cancer.

## Data Availability

All data and materials supporting the conclusions were included in this paper. More details are available on request.

## Abbreviations

3′-UTR: 3′-untranslated regions
EMT: Epithelial to mesenchymal transition
LncRNA: Long non-coding RNA
miR-513: microRNA-513
MUT: Mutant type
NC: Negative control
NSG: NOD-SCID IL-2receptor gamma null
OD: Optical density
OSC: Ovarian serous cancer
qRT-PCR: Quantitative reverse-transcription polymerase chain reaction
siRNA: Small interfering RNA
YAP1: Yes-associated protein 1
